# Prospects for a novel ultrashort pulsed laser technology for pathogen inactivation

**DOI:** 10.1186/1423-0127-19-62

**Published:** 2012-07-06

**Authors:** Shaw-Wei D Tsen, Tzyy Choou Wu, Juliann G Kiang, Kong-Thon Tsen

**Affiliations:** 1 Department of Radiology, Washington University School of Medicine, St. Louis, MO, 63110, USA; 2 Departments of Pathology, Johns Hopkins School of Medicine, Baltimore, MD, 21231, USA; 3 Departments of Oncology, Johns Hopkins School of Medicine, Baltimore, MD, 21231, USA; 4 Obstetrics and Gynecology, Johns Hopkins School of Medicine, Baltimore, MD, 21231, USA; 5 Molecular Microbiology and Immunology, Johns Hopkins School of Medicine, Baltimore, MD, 21231, USA; 6 Scientific Research Department, Armed Forces Radiobiology Research Institute, 8901 Wisconsin Avenue, Bethesda, MD, 20889-5603, USA; 7 Department of Medicine, Uniformed Services University of the Health Sciences, 4301 Jones Bridge Road, Bethesda, MD, 20889-5603, USA; 8 Department of Radiation Biology, Uniformed Services University of the Health Sciences, 4301 Jones Bridge Road, Bethesda, MD, 20889-5603, USA; 9 Department of Physics, Arizona State University, Tempe, AZ, 85287, USA

## Abstract

The threat of emerging pathogens and microbial drug resistance has spurred tremendous efforts to develop new and more effective antimicrobial strategies. Recently, a novel ultrashort pulsed (USP) laser technology has been developed that enables efficient and chemical-free inactivation of a wide spectrum of viral and bacterial pathogens. Such a technology circumvents the need to introduce potentially toxic chemicals and could permit safe and environmentally friendly pathogen reduction, with a multitude of possible applications including the sterilization of pharmaceuticals and blood products, and the generation of attenuated or inactivated vaccines.

## Review

Despite the myriad antimicrobial methods that have been developed to combat infectious disease, microbial pathogens continue to evolve and acquire resistance. In addition, emerging pathogens such as Human Immunodeficiency Virus (HIV) [1] in the 1980s and more recently West Nile Virus (WNV) [2] continue to pose threats before testing and containment strategies are in place. Therefore, new and more effective pathogen inactivation strategies are urgently needed.

Use of Ultrashort pulsed (USP) lasers for selective disinfection has emerged as a potentially attractive antimicrobial strategy. USP laser treatment has been shown to inactivate a variety of viruses including HIV, Influenza virus, Human Papillomavirus (HPV), Murine Noroviruses, Hepatitis A Virus (HAV), Encephalomyocarditis Virus (EMCV), Tobacco Mosaic Virus (TMV) and M13 bacteriophage, as well as bacteria such as *E. coliSalmonella* spp, and *Listeria*[3-11].

The USP laser technology has the following advantages over the current methods of disinfection of pathogens:

(1) With conventional pharmaceutical antiviral and antibacterial treatments, a new drug is usually required to combat new or mutated strains of microorganisms. In contrast, the USP laser method is effective for the inactivation of enveloped and non-enveloped, single-stranded, double-stranded DNA, RNA viruses, and gram-positive and gram-negative bacteria [3-11], suggesting that the USP laser technique could represent a general method for inactivating viral and bacterial pathogens regardless of their structural composition or mutation status. For the inactivation of a virus, the USP laser method excites mechanical vibrations of the capsid of a virus and targets the weak links of the viral protein coat, leading to its loss of infectivity; for the inactivation of a bacterium, the USP laser technique relaxes the super-coiled double-stranded DNA causing damage and subsequent death of the bacterium. This is demonstrated by the results in Table 1[3-11] in which a variety of viruses and bacteria have been shown to be efficiently inactivated by the USP lasers.

**Table 1:** Killing efficacy for a variety of microorganisms using A 425 nm- femtosecond pulsed laser (laser exposure time = 3.6 seconds)

**Microorganism**	**Properties**	**Load reduction**
**Human Immunodeficiency Virus (HIV)**	Enveloped, single-stranded RNA	10^4^
**Influenza Virus**	Enveloped, single-stranded RNA	10^5^
**Encephalomyocarditis virus (EMCV)**	Non-enveloped, single-stranded RNA	10^3^
**Murine norovirus (MNV)**	Non-enveloped, single-stranded RNA	10^3^
**Hepatitis A virus (HAV)**	Non-enveloped, single-stranded RNA	10^3^
**Human Papillomavirus (HPV)**	Non-enveloped, double-stranded DNA	10^5^
**M13 bacteriophage**	Non-enveloped, single-stranded DNA	10^5^
**Escherichia coli**	Gram negative	10^4^
**Salmonella typhi**	Gram negative	10^5^
**Listeria monocytogenes**	Gram positive	10^3^
**Enterobacter Sakazakii**	Gram negative	10^3^

(2) Existing disinfection methods such as irradiation of ultraviolet (UV) light, gamma-ray, UV/photochemicals, microwave absorption, and pharmaceutical antiviral and antibacterial treatments are not selective; as a result, severe side effects may accompany the treatments. On the other hand, the USP laser method has been shown [3,6,9] to inactivate undesired microorganisms like viruses and bacteria while leaving desired materials such as mammalian cells and proteins unharmed; i.e., the USP laser technique is capable of selective disinfection and therefore has minimal potential side effects. Table 2 shows experimental results on the selectivity of a near-infrared USP laser on a variety of microorganisms. The intriguing feature worthwhile mentioning is that there exists a therapeutic window in laser power density between 1 GW/cm^2^ and 10 GW/cm^2^ which allows the inactivation of a variety of pathogens while leaving mammalian cells unharmed. The existence of this window enables selective inactivation of microorganisms.

(3) Because of the nature of USP laser inactivation, the USP laser technique is sensitive to the global oscillation of the capsid but not to minor changes caused by nucleic acid mutation in the pathogens; as a result the USP laser technology can be used to inactivate both wild-type and mutated/drug-resistant strains of microorganisms. An example is given for M13 bacteriophages in which both wild-type and engineered strains are efficiently inactivated by the irradiation of USP lasers [9]. This intriguing feature makes the USP laser technique particularly suitable for the disinfection of rapidly evolving or drug-resistant viral and bacterial species such as HIV and MRSA, respectively.

(4) Currently available pathogen reduction methods for blood components usually involve the addition of potentially toxic or carcinogenic chemicals. Residual amounts of these chemicals can remain within the transfusion products and then be transfused. In addition, it is likely that in some cases these chemicals may interact with the product itself, potentially altering its structure or function. The potential side effects due to the introduction of such chemicals during the pathogen reduction process is a major concern from the FDA standpoint [12] On the other hand, the USP laser technology is chemical-free; in other words, it does not involve introducing chemicals during pathogen reduction. This makes the USP laser method safe and environmentally friendly, and advantageous for treating products such as blood products, pharmaceuticals, therapeutics, vaccines, and other agents that are used in humans.

**Table 2:** Threshold laser power density for inactivation of viruses and cells

	**Viruses and Cells**						
	**M13**	**TMV**	**HPV**	**HIV**	**Human red blood cell**	** Human Jurkat T-cell**	**Mouse dendritic cell**
Threshold Laser Power Density for inactivation(GW/cm^2^)	0.06	0.85	1.0	1.1	15	22	12

## Basic mechanism of inactivation of pathogens by ultrashort pulsed lasers

### Inactivation of a virus by ultrashort pulsed lasers

**Figure 1:**
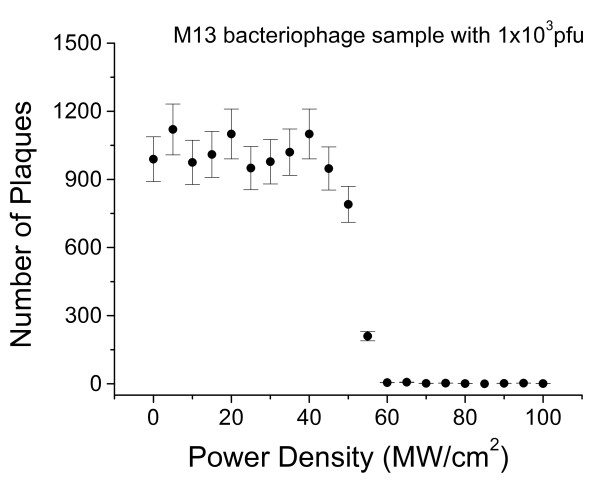
**Number of pfu as a function of laser power density for M13 bacteriophages excited by a near-infrared Ti-sapphire cw mode-locked laser.** See text for discussions.

We take M13 as an example for demonstration. Figure 1 shows plaque forming units (pfu) as a function of laser power density for M13 bacteriophages excited by a near-infrared Ti-sapphire cw mode-locked laser [4,5,7] The intriguing feature of these assay results is the rapid cut-off of the pfu of M13 bacteriophages at around 60 MW/cm^2^. A similar feature (which is not shown here) is also found when a visible USP laser is used for inactivation. This unique feature of inactivation upon laser power density indicates the emergence of a new virus inactivation mechanism for M13 bacteriophages by the irradiation of USP lasers – impulsive stimulated Raman scattering (ISRS) – which is elucidated below.

**Figure 2:**
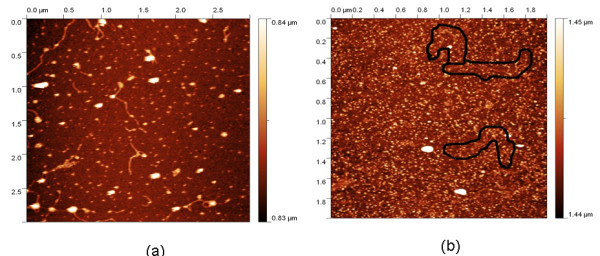
**Atomic Force Microscope images of M13 bacterioaphages (a) without laser irradiation and (b) with laser irradiation by a visible femtosecond laser.** For clarity, the black curves in (**b**) were drawn to encircle the bare DNAs. See text for discussions (with publisher’s permission).

The atomic force microscope (AFM) images from the control and laser treated M13 bacteriophage samples provide an important clue for the inactivation mechanism. The AFM images of a M13 bacteriophage sample before and after the visible USP laser irradiation are shown in Figure 2(a) and 2(b), respectively [10]. The relatively smooth worm-like features having a diameter of about 6 nm and about 850 nm in length in Figure 2(a) revealed the presence of M13 bacteriophages in the control. Figure (b) showed, in contrast to Figure 2(a), the appearance of many small structures which were about 6 nm in diameter after laser irradiation. As discussed later, these small structures were consistent with the size of individual α-helix protein units of which the protein capsid of the M13 bacteriophage is composed. As a result, these small structures are attributed to individual α-helix protein units of the M13 bacteriophage. In addition, some zigzagged worm-like features (encircled by artificially drawn black curves for the sake of clarity) were observed. The fact that its length was about 850 nm and that it was in a zigzagged structure indicated that these zigzagged structures were naked viral genomic DNAs from M13 bacteriophages. The observation of the naked DNAs in the laser-irradiated M13 bacteriophage sample indicated that irradiation of the visible USP laser severely altered the structural integrity of the protein shell of the M13 bacteriophages, potentially causing the DNA to “leak out”.

By taking into account the size of small structures about 6 nm in diameter in the AFM images of M13 bacteriophages after USP laser irradiation in Figure 2(b), the resolution of the tip of AFM used in the imaging, and the actual size of the α-helix protein unit which forms the capsid of a M13 bacteriophage, we have found that the small structures observed in Figure 2(b) are consistent in size with those of the α-helix protein units of the capsid of M13 bacteriophages. This analysis further supports our conclusion that USP laser irradiation under our experimental conditions does not damage individual protein units in M13 bacteriophages.

**Figure 3:**
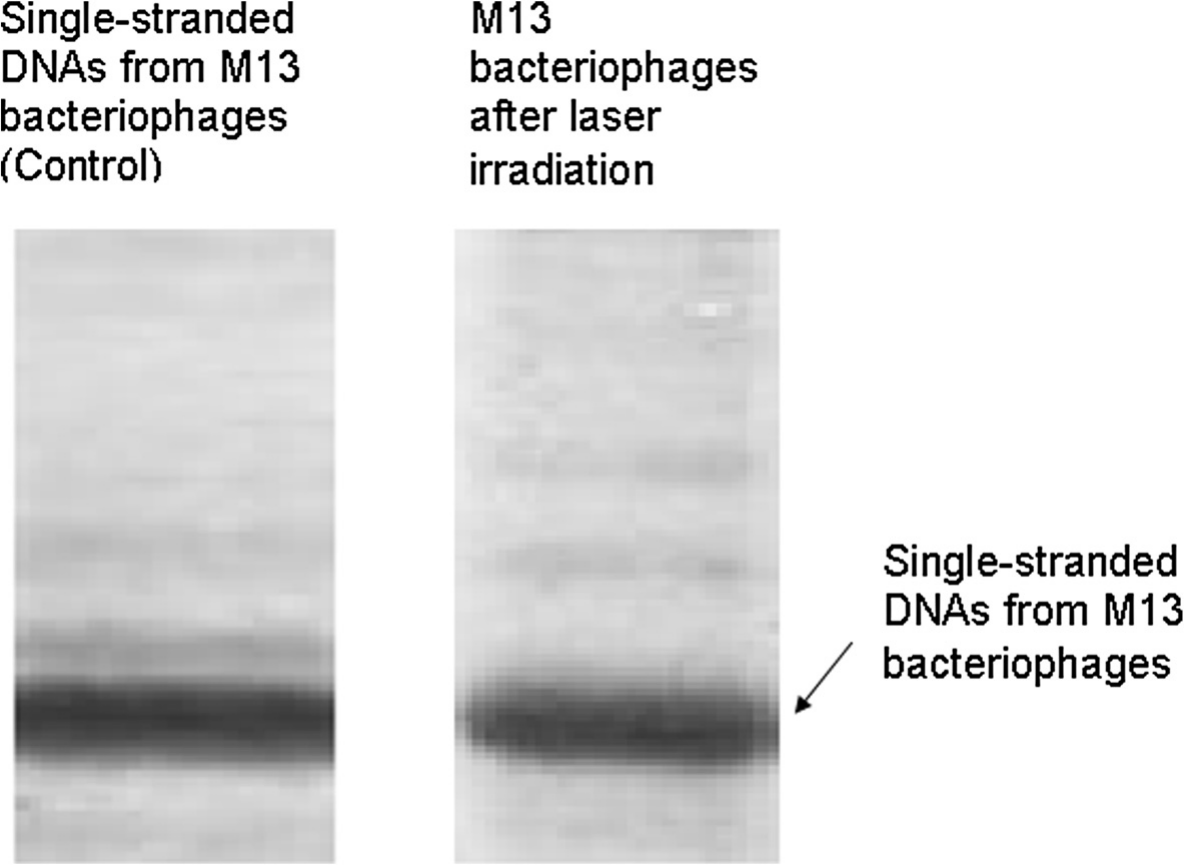
**Gel electrophoresis experiments on single-stranded DNAs of M13 bacteriophages (control) and the laser-irradiated M13 bacteriophages after treatment with the visible femtosecond laser, operated at 425 nm, at a repetition rate of 80 MHz, average power of 100 mWs, laser spot size of about 100 micron, and laser irradiation for 1 hr.** For clarity, on the laser irradiated sample, an additional band resulting from the α-helix protein units of M13 bacteriophages, which appears on a different scale, is not shown (with publisher’s permission).

Figure 3 shows the result from agarose gel electrophoresis on single-stranded DNAs from M13 bacteriophages (control) and from M13 bacteriophages irradiated with a visible USP laser [10]. The laser-irradiated M13 bacteriophage sample showed a single dark band similar in width to and located at the same position as that of the control sample. Therefore, these experimental results indicated that, within experimental uncertainty, irradiation of a visible USP laser caused no severe structural change of single-stranded DNAs of M13 bacteriophages. In other words, the gel electrophoresis results of Figure 3 on the single-stranded DNAs of M13 bacteriophages indicate that irradiation of a visible USP laser does not significantly alter the structure of single-stranded DNA.

**Figure 4:**
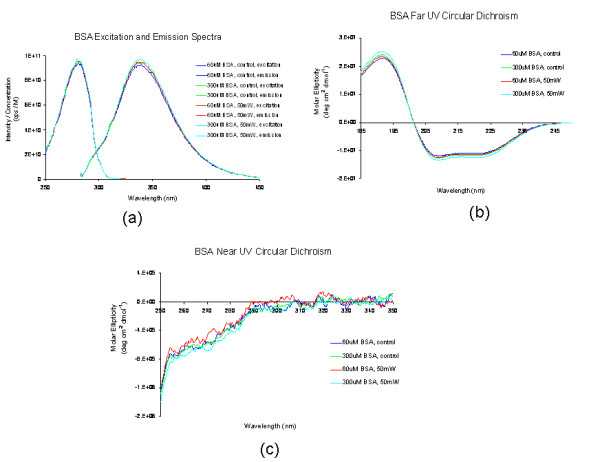
(a): Excitation and luminescence spectra of BSA proteins; (b): Far UV circular dichroism spectra of BSA proteins; (c): Near UV circular dichroism spectra of BSA proteins (with publisher’s permission).

The luminescence, excitation, and circular dichroism (CD) spectra from amino acids of proteins are very sensitive to the structural changes of proteins. Therefore, these optical characterization methods were employed to detect the primary and secondary structural changes of proteins before and after the visible USP laser irradiation. Figures 4(a)4(b)4(c) show our preliminary results for bovine serum albumin (BSA) proteins in buffer solution with and without irradiation with an USP laser [10]. In Figure 4(a), the excitation spectrum corresponded to the broad structure centered around 280 nm. The luminescence spectrum represented the broad peak around 340 nm. Each spectrum contained 4 curves in which two of them were control and two were laser-irradiated samples, as indicated. The two control samples and two laser-irradiated samples had 60 μM, 300μM of BSA proteins, respectively. For clarity, the spectra shown were normalized to the concentration of BSA proteins. In Figure 4(b), the far UV CD contained four curves, in which two of them were control and two were laser-irradiated samples. The two control samples and two laser-irradiated samples had 60μM, 300μM of BSA proteins, respectively. For clarity, the spectra shown were normalized to the concentration of BSA proteins. In Figure 4(c), the near UV CD included four curves in which two of them were control and two were laser-irradiated samples. The two control samples and two laser-irradiated samples had 60 μM, 300 μM of BSA proteins, respectively. For clarity, the spectra shown were normalized to the concentration of BSA proteins. The experimental results show that, within experimental uncertainty, the luminescence, excitation spectra and circular dichroism of BSA proteins remained practically the same before and after the laser irradiation, indicating minimal or no structural changes in BSA proteins after irradiation with a visible USP laser. Therefore, these experimental results on the optical characterization of BSA proteins suggest that there is virtually no structural change in BSA proteins upon USP laser irradiation. Because BSA is primarily made up of α-helix proteins, and the capsid of a M13 bacteriophage is mostly composed of α-helix protein units, these results suggest that the visible USP laser irradiation will not damage the individual protein units that comprise the protein capsid of M13 bacteriophage.

Thus, the AFM images of Figure 2 together with the DNA gel electrophoresis results of Figure 3 and optical results of BSA proteins of Figure 4 are consistent with our model: that irradiation with a USP laser alters the structural integrity of the protein capsid of M13 bacteriophages by disrupting weak interactions *between* proteins without damaging either the viral genomic single-stranded DNA or the individual protein units of M13 bacteriophage capsid.

Irradiation with an intense ultrashort pulsed laser such as a femtosecond laser can deposit laser energy onto the protein capsid of a viral particle by the excitation of low-frequency acoustic vibrations on the capsid of a virus. This process, known as impulsive stimulated Raman scattering (ISRS), has been used to deposit laser energy to solid state systems as well as to biological molecules [13-20].

The ISRS process can be understood as follows:

The vibrational mode of a macromolecule such as a virus excited by the laser is represented by normal coordinate Q. If we ignore dispersion in the index of refraction and assume that the incident electric field from the excitation laser is not depleted by the stimulated scattering, the equation of motion for Q can be written as [21,22]

(1) $$\frac{\partial^{2}Q}{\partial t^{2}}+2\gamma \frac{\partial Q}{\partial t}+\omega_{0}{}^{2}Q=f(t)$$

where$\omega_{0}$ is the angular frequency of vibration,  is the damping constant and f(t) is the impulsive driving force produced by the excitation laser and is described next.

The electric field ${\vec{E}}_{L}$ of the laser induces a polarization on the molecule due to its polarizability *α* as $\vec{P}=\alpha {\vec{E}}_{L}$, where for simplicity we neglect the tensor properties of *α*. The polarizability has a static part that produces elastic Rayleigh scattering, and a part that is modulated by the oscillating displacement Q. It is this modulated contribution that produces the Raman effect and the ISRS process in the macromolecule. The polarizability *α*, expanded in a Taylor series in Q, is

(2) $$\alpha (Q)=\alpha_{0}+\alpha_{0}{}^{'}Q+\frac{1}{2}\alpha_{0}{}^{''}Q^{2}+$$

higher order terms in Q (2); where α0 is the zero order term $\alpha_{0}{}^{'}Q\equiv {\left(\frac{\partial \alpha }{\partial Q}\right)}_{0}Q$ is the first order term resulting in the first order Raman scattering processes; $\frac{1}{2}\alpha_{0}{}^{''}Q^{2}\equiv \frac{1}{2}{\left(\frac{\partial^{2}\alpha }{\partial Q^{2}}\right)}_{0}Q^{2}$ is the second order term, etc.

The potential energy stored in an induced polarization is $U(Q,t)=-\frac{1}{2}\vec{P}(Q,t)\cdot {\vec{E}}_{L}(t)$. If we keep up to the first order term and neglect the second order and higher order terms in the polarization expansion in Eq, (2), the generalized driving force $f(t)=-\frac{\partial U(Q,t)}{\partial Q}$ on the right hand side of Eq. (1) becomes

(3) $$f(t)=\frac{1}{2}\alpha_{0}^{'}E_{L}^{2}$$

Equation (1) with f(t) given by Eq. (3) can be solved by using Green’s function method to determine the normal coordinate Q(t) [13,23]. In particular, for excitation by a single-beam ultrashort laser having a pulse width of $\tau_{L}$, and intensity$I(t)=I_{0}\cdot e^{-(t^{2}/\tau_{L}^{2})}$, assuming small damping, the displacement is $Q(t)=Q_{0}e^{-\gamma t}\sin(\omega_{0}t).$ Of greatest importance in $Q(t)=Q_{0}e^{-\gamma t}\sin(\omega_{0}t)$ is the amplitude $Q_{0}$ of the displacement away from the equilibrium position of the molecule produced by ISRS process, which is given by [13,23]

(4) $$Q_{0}=\frac{\sqrt{\pi }}{2}\frac{n}{cK\epsilon_{0}}\alpha_{0}^{'}\frac{\tau_{L}}{\omega_{0}}\cdot I_{0}\cdot e^{-(\omega_{0}{}^{2}\tau_{L}{}^{2}/4)}\text{.}$$

Here $I_{0}$ is the peak intensity of the excitation laser, $\alpha_{0}^{'}$ is the polarizability derivative proportional to the amplitude of the Raman scattering cross section, *n* is the index of refraction, c the speed of light, and $K\epsilon_{0}$ the permittivity of the dielectric medium.

Therefore, in this ISRS process, the deposited laser energy on the protein capsid of a viral particle is proportional to the square of the laser intensity and to the Raman scattering cross section. If the deposited laser energy or the amplitude of the excited resonance mode on the capsid of a viral particle is large enough, it can break the weak links (for example, hydrogen bonds or hydrophobic contacts) between the proteins, damage to the capsid of the virus occurs, leading to the viral inactivation.

In the ISRS process, operated in near-infrared/visible wavelength range to which water is transparent, one way of selective killing of microorganisms is by varying the laser power density; the other way of selective killing of microorganisms in biological systems is by controlling the range of spectral content of an ultrashort pulsed laser. For a transform-limited pulsed laser, by using Heisenberg uncertainty principle, it is equivalent to controlling the laser pulse width. The presence of the factor $e^{-\omega_{0}{}^{2}\tau_{L}{}^{2}/4}$ in Eq. (4) indicates that in order to excited significantly large amplitude $Q_{0}$ of a vibrational frequency $\omega_{0}$ in a microorganism for damaging effect, the excitation laser pulse width $\tau_{L}$ has to be chosen so that $\omega_{0}\tau_{L}$≤1. Because each microorganism has its own characteristic resonance vibrational frequency $\omega_{0}$, by choosing the proper pulse width of an ultrashort pulsed laser, the amplitude of this resonance mode can be excited so high as to damage and inactivate the microorganism.

We note that cw (continuous wave) laser cannot excite the resonance mode $\omega_{0}$ of a microorganism through an ISRS process. Because $\tau_{L}=\infty$ for a cw laser, Eq. (4) therefore indicates that the amplitude of the excited vibrational mode is zero. A Q-switched laser cannot excite the resonance mode $\omega_{0}$ of a typical microorganism through ISRS process either. This is because each microorganism has a characteristic resonance vibrational frequency $\omega_{0}$ which typically is in the range of 100 GHz;[24-29] for example, helix-shaped M13 bacteriophage is around 300 GHz [27-29] and icosahedral viruses of 30 nm in size like murine norovirus is around 65 GHz [24] and if we use a viral frequency of 100GHz and the fact that a typical Q-switched laser has a pulse width of about 100 nanosecond, from Eq. (4), the factor $e^{-(\omega_{0}{}^{2}\tau_{L}{}^{2}/4)}$ becomes vanishingly small. Therefore, the amplitude of vibrations a Q-switched laser will excite is negligibly small.

The rapid switch from non-inactivation to inactivation at the laser power density of 60 MW/*cm*^2^ shown in Figure 1 for M13 bacteriophages can be explained by the ISRS process. When the laser power density is small (<60*MW*/*cm*^2^), the excited amplitude of vibration on the capsid of M13 bacteriophage is not large enough to break the weak links and no inactivation is observed; however, as the laser power density increases to and beyond *60 MW/cm*^*2*^, the excited amplitude of vibration becomes large enough to break the weak links on the capsid of the M13 bacteriophage, leading to the inactivation of M13 bacteriophage.

To further support our argument that viral particles are inactivated by the irradiation of USP lasers through an ISRS process, we show experimental results of the inactivation of M13 bacteriophages as a function of laser pulse widths/spectral widths in Table 3[4-7] while the laser intensity is kept constant. The abrupt change from inactivation to no inactivation observed in the experiments when the pulse width of the laser changes from 500 fs to 800 fs is consistent with the prediction of Eq. (4) by using the Raman mode frequency of 10*cm*^−1^which was measured by Raman spectroscopy for M13 bacteriophages [27-29].

**Table 3:** Dependence of the status of M13 bacteriophage on laser pulse width

Pulse Width (fs)	80	250	500	800	1000
Spectral Width (cm^–1^)	(80)	(25)	(12)	(6.5)	(5)
Status Inactivation (Yes or No)	Yes	Yes	Yes	No	No

(The excitation laser intensity is kept at 5.6 × 10^–6^J/cm^2^).

(The numbers with in the brackets indicate the spectral width in cm^–1^).

**Figure 5:**
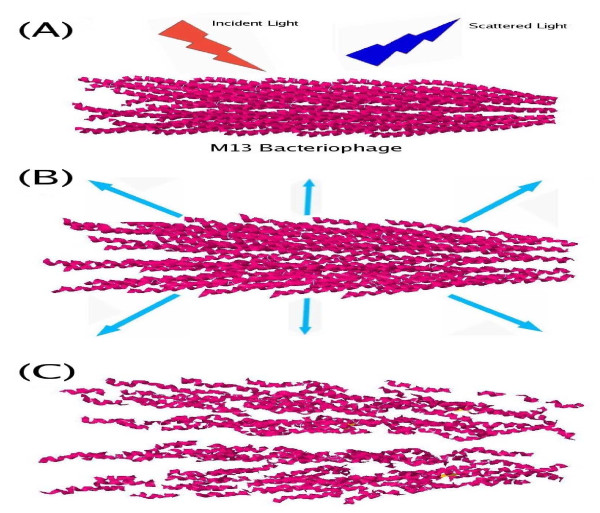
**Diagrams showing how the M13 bacteriophage is inactivated by an USP laser.** (**A**) The electric field from a femtosecond laser produces an impulsive force through the induced charge polarization on the virus; (**B**) The resultant mechanical impact coherently excites Raman-active vibrational modes on the capsid of the virus; (**C**) If the pulse width/spectral width and intensity of the USP laser are appropriately chosen, the vibrational modes can be excited to such high energy states as to break off the weak links between proteins in the capsid of the virus, damaging/disintegrating the capsid and leading to the inactivation of the virus.

Therefore, schematically, this is what is happening in our model for USP laser inactivation of viruses such as the M13 bacteriophage: The electric field from a femtosecond laser produces an impulsive force through the induced charge polarization on the virus, as shown in Figure 5(A). This mechanical impact coherently excites Raman-active vibrational modes on the capsid of the virus, as depicted in Figure 5(B). Figure 5(C) demonstrates that if the pulse width/spectral width and intensity of the USP laser are appropriately chosen, the vibrational modes can be excited to such high energy states as to break off the weak links on the capsid of the virus, damaging/disintegrating the capsid and leading to the inactivation of the virus.

### Inactivation of bacteria by ultrashort pulsed lasers

**Figure 6:**
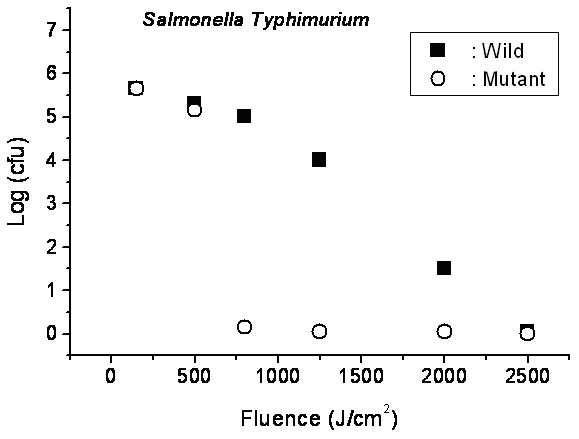
**Log-kill factor as a function of laser fluence for the wild, mutant *Salmonella typhimurium,* as indicated (with publisher’s permission).**

We take *Salmonella typhimurium* as an example*.* To obtain insight into the inactivation mechanisms, we have performed inactivation of a mutant *Salmonella typhimurium* by a visible USP laser. The mutant is deficient in RecA proteins which are responsible for the repair of damaged DNA. In other words, the mutant is very sensitive/vulnerable to the damage of DNA. Figure 6[10] shows the inactivation of both the wild-type and mutant *Salmonella typhimurium* by a visible USP laser as a function of the laser fluence. In general, the log – load reduction factor at a given laser dose has be found to be higher for the mutant than for the wild strain. In particular, our experimental results indicate that by using the USP laser, with laser dose of about 800 *J/cm*^*2*^, a log - load reduction factor of about 5 for mutant *Salmonella typhimurium* was observed; however, by employing the same laser parameter, a log-kill factor of only 0.5 for the wild *Salmonella typhimurium* was found. Because the only difference between these two strains of *Salmonella typhimurium* is the RecA proteins which are in charge of the repair of damaged DNA, these experimental results indicate that irradiation of a visible USP laser causes DNA damage and subsequent inactivation of the *Salmonella typhimurium*.

**Figure 7:**
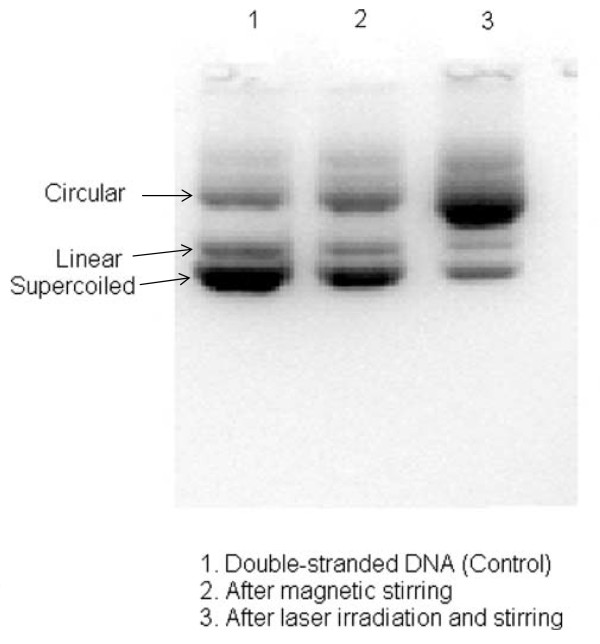
**Gel electrophoresis experiments on double-stranded DNAs.** #1 is the control without magnetic stirring showing the presence of super-coiled, linear and circular DNAs; #2 is another control with magnetic stirring; #3 is the laser-irradiated sample with magnetic stirring. The visible femtosecond laser is operated at 425 nm, at a repetition rate of 80 MHz, with an average power of 100 mWs, laser spot size of about 100 micron, and laser irradiation time of 1 hr.

Figure 7 demonstrates our preliminary results for isolated double-stranded DNAs in buffer solution before and after irradiation by a visible femtosecond laser, as detected by the agarose gel electrophoresis method [10]. The control sample (labeled No. 1) revealed the presence of three dark bands corresponding to circular, linear, and super-coiled double-stranded DNA, respectively. Sample No. 2 showed that stirring the sample slightly changed the relative darkness of the bands. On the other hand, the laser-irradiated sample (labeled No. 3) showed that the relative darkness of the three bands was greatly altered. These data suggest that the effects of visible femtosecond laser irradiation primarily caused relaxation of the supercoiled double-stranded DNA to produce relaxed circular double-stranded DNA. Because forced changes in the supercoiling status of DNA can disrupt cellular metabolism, which can lead to the death of the cell, one mechanism which can contribute to the inactivation of *Salmonella typhimurium* by the irradiation of a visible USP laser is relaxation of supercoiled DNA in the bacteria.

It has been known that photo-stimulation of endogenous intracellular porphyrin molecules in the bacteria by continuous wave visible light irradiation may result in the production of reactive oxygen species (ROS), predominantly singlet oxygen, and consequently, damage to the DNA and the death of bacteria [30-35]. Therefore, the other mechanism which can contribute to the inactivation of *Salmonella typhimurium* by a visible USP laser is the photo-production of ROS.

## Prospects of the selective disinfection of pathogens by USP lasers

In the following sections, we discuss a few of the potential applications we envision for this USP laser technology.

### Decontamination of blood products for transfusion

Millions of red blood cell, platelet, plasma and coagulation factor transfusions are performed every year in the United States alone. Implementation of specific donor screening criteria together with nucleic acid and immunologic testing have significantly reduced the risk of transmission of blood components through transfusion for a number of pathogens. This system, however, does not solve all problems posed by pathogens. This is because (1) not all recognized threats have been adequately addressed; (2) there exists a “window period” for a donor during which the infection cannot be detected by testing but during which the donor may be infectious; and (3) screening and tests can only be performed for those pathogens that have been recognized and for which tests are available. Unknown/emerging pathogens will remain as a threat as evidenced by the emergence of HIV and WNV in the past [36]. Therefore, from the transfusion recipient’s viewpoint, the ideal strategy for ensuring transfusion safety of blood components should be to implement a preemptive pathogen reduction (PR) technology, which can universally eliminate microbes in a blood product without chemicals and without adversely affecting the function of the blood product itself. For details of all the currently available PR techniques for the disinfection of blood components, please refer to [37-42]. PR technique in plasma components are dominated by solvent detergent treatment [43], methylene blue method [44] and UV-activated photochemical method [45-47] such as using amotosalen and riboflavin. Although these are effective in pathogen reduction, some concerns still exist. Several PR treatments have been developed for platelets. Because these treatments share the use of UV light, although at different wavelengths, possible damage to the blood product and/or microbial resistance becomes a concern. Techniques for PR in red blood cells are largely still under development. A significant concern of the above-mentioned techniques is the addition of foreign chemicals which cannot be completely removed after the treatments. These residual chemicals may have short or long term adverse effects on patients who require frequent transfusion of blood components.

In contrast, the chemical-free USP laser technology has been shown to kill 3–5 log_10_ of a variety of pathogens (see Table 1), and more importantly, it exhibits selectivity for microbes over desirable proteins and mammalian cells (see Table 2). Therefore, the USP laser technology represents a plausible pathogen inactivation technology for pathogen reduction of blood products.

### Sterilization of biologicals and pharmaceuticals

Biologicals and pharmaceuticals used in the clinic as well as reagents or cell cultures used in research laboratories can be contaminated with microbes such as *Mycoplasma spp.*, viruses and bacteria, which can affect their safety profile and their biological function. Traditionally, enveloped viruses or bacteria can be killed by the addition of detergent or alcohol-based chemicals. Non–enveloped viruses are harder to kill and are usually inactivated by either heating or using bleach; however, either the heating process or the addition of such chemicals raises the concern of potential side effects. Filtration is an effective way of removing pathogens; however, it is not applicable when the size of undesired pathogen(s) is comparable to that of the desired product. In these cases, a technique that can non-invasively sterilize a solution containing a desired reagent, cell culture, or pharmaceutical without changing the product’s structure or function is desirable.

In this regard, USP laser technology represents a plausible method for accomplishing sterilization of biologicals, pharmaceuticals, cell cultures, and reagents. Our preliminary results suggest that a visible USP laser can be used to inactivate viral particles and bacteria, without altering the structure of individual protein units [10]. Therefore, USP laser technology could conceivably be useful for sterilizing biologicals, pharmaceuticals, cell cultures, and reagents.

### Generation of efficient and safe vaccines

The use of killed or attenuated whole microorganisms is an attractive strategy for the development of immunogenic vaccines for many diseases including tuberculosis and malaria [48]. Whole organism vaccines include most of the relevant antigens and retain many of the immunostimulatory components necessary to induce a strong and specific immune response. Various techniques have been applied to this end, including chemical killing, [49] UV/psoralen treatment [48] and gamma-ray irradiation [50]. Chemical methods such as the application of formalin have the advantages of being simple and cost effective; however, it is not as efficient as other methods. Furthermore, the addition of chemicals raises concerns of potential side effects. UV/psoralen treatment has been shown to be promising in generating killed but metabolically active pathogen vaccines in mouse models; however, the added chemicals are very difficult to remove completely. This raises the concern of potential adverse effects when applied in the clinic. Gamma ray irradiation has been demonstrated to be effective in generating inactivated vaccines in mouse models; however, the gamma-ray photon is high-energy ionizing radiation which will break any chemical bonds in its path including covalent, ionic, and hydrogen bonds in the microorganism. As a result, the use of gamma-ray treated vaccines raises concerns that “new chemical species” may be created that may have adverse effects in humans.

We envision that the use of USP lasers to generate whole inactivated vaccines could be advantageous over current methods, partly because the technique kills the organism efficiently with potentially minimal changes to antigenic and/or immunostimulatory structures, [3-10] and partly because no potentially toxic chemicals are added or created. As a matter of fact, our preliminary results (not shown here) with a USP laser-inactivated H1N1 flu vaccine demonstrates vaccine-induced T-cell responses and protection against challenge in a mouse model.

## Potential experimental layout

**Figure 8:**
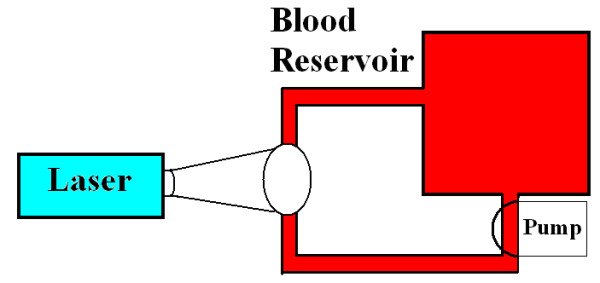
Potential experimental setup for use of USP laser technique in pathogen reduction of blood products.

One possible approach of using the USP laser technology for selective PR of blood components and pharmaceuticals, and for vaccine production described above is to use a syringe pump to channel the samples through narrow tubing for laser irradiation (see Figure 8).

**Figure 9:**
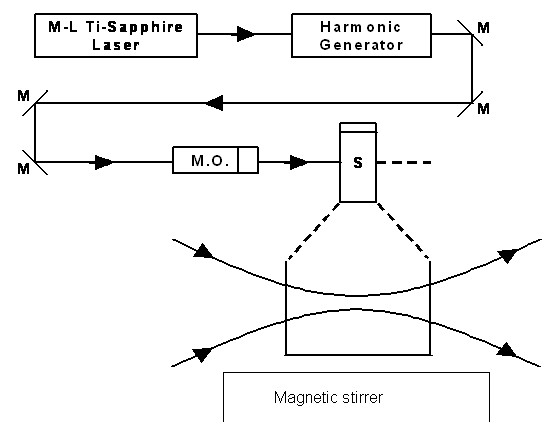
Potential experimental set up for the inactivation of viral particles and bacteria with an USP laser. M.O.: focusing lens; M: mirror; S: vial containing viruses/bacteria in buffer solutions.

If an intense USP laser system is available, an alternative experimental setup involving a magnetic stirrer such as that in Figure 9 can be used.

## Conclusion

The emergence of drug-resistant microbes and new, heretofore-unknown pathogens has renewed the search for effective antimicrobial technologies. The recently developed USP laser technique for microbial load reduction could represent a universal, non-invasive, and environmentally friendly method for selective inactivation of microbes without the use of clinically toxic or environmentally damaging agents. We predict that the USP laser technology will be used for (1) Decontamination of blood products for transfusion; (2) Sterilization of biologicals, pharmaceuticals, cell cultures, and reagents; and (3) Generation of efficient and safe vaccines in the near future.

## Competing interests

The authors declare that they have no competing interests.

## Authors’ contributions

SWDT proposed the idea of pathogen inactivation by ultrashort pulsed lasers, performed laser irradiation experiments, carried out the assays and drafted the manuscript. TCW participated in the assays and discussions. JGK participated in the assay and discussions. KTT proposed the idea of pathogen inactivation by ultrashort pulsed lasers, performed laser irradiation experiments, and drafted the manuscript. All authors read and approved the final manuscript.
